# The regulatory mechanisms and translational applications of non-coding RNA in SARS-CoV-2 infection-related cardiovascular pathology

**DOI:** 10.3389/fcvm.2026.1837477

**Published:** 2026-07-15

**Authors:** Ziwei Cao, Yurong Song, Jing Li, Yuan Cao

**Affiliations:** 1 School of Medical Laboratory Science, Shandong Second Medical University, Weifang, Shandong, China; 2 Department of Basic Medical Sciences, The 960th Hospital of PLA, Jinan, Shandong, China; 3 State Key Laboratory of Pathogen and Biosecurity, Academy of Military Medical Sciences, Beijing, China

**Keywords:** biological marker, cardiac injury, cardiovascular complication, COVID-19, non-coding RNA, SARS-CoV-2, therapeutic target

## Abstract

SARS-CoV-2 infection not only leads to severe respiratory diseases but also has a significant impact on the cardiovascular system, inducing acute cardiac injury and various long-term cardiovascular complications. Non-coding RNAs (ncRNAs), as key molecules in gene expression regulation, exhibit important regulatory roles in viral infections and cardiovascular pathological processes. Current research indicates that ncRNAs such as miRNA, lncRNA, and circRNA participate in the occurrence and development of cardiac injury related to SARS-CoV-2 infection by regulating apoptosis, metabolic reprogramming, and cell communication. However, there are still many challenges and unresolved mysteries regarding their specific regulatory networks and clinical applications. This review systematically summarizes the expression changes and functional characteristics of ncRNAs in COVID-19-related cardiovascular diseases, focusing on their potential as biomarkers and therapeutic targets, and combining the latest multi-omics data and cutting-edge technologies to anticipate the development direction of precise interventions and personalized treatments.

## 1 Introduction

The global pandemic caused by severe acute respiratory syndrome coronavirus 2 (SARS-CoV-2), the etiological agent of coronavirus disease 2019 (COVID-19), has resulted in unprecedented morbidity and mortality worldwide (1). While primarily recognized as a respiratory illness, it is now unequivocally established that COVID-19 exerts profound and potentially life-threatening effects on the cardiovascular system (2–4). Clinical data consistently reveal that myocardial injury, evidenced by elevated cardiac troponin levels, is present in over 20%–25% of hospitalized patients and is associated with a worse prognosis and mortality rates approaching 40% (5–7). The spectrum of cardiovascular complications in COVID-19 is broad and severe, encompassing acute coronary syndromes, myocarditis, arrhythmias, heart failure, Takotsubo cardiomyopathy, and a heightened risk of venous and arterial thromboembolic events (2, 3, 8, 9). This bidirectional relationship is further complicated by the observation that patients with preexisting cardiovascular comorbidities are not only more susceptible to severe COVID-19 infection but also face increased risk of further cardiac complications and higher mortality (10–12). The pervasive nature of this interplay underscores the critical need to unravel the complex pathophysiological mechanisms linking SARS-CoV-2 infection to cardiovascular damage.

The pathogenic mechanisms underlying COVID-19-associated cardiovascular injury are multifactorial and involve a convergence of direct viral cytotoxicity and indirect systemic effects (9). SARS-CoV-2 gains cellular entry primarily via the angiotensin-converting enzyme 2 (ACE2) receptor, which is expressed on cardiomyocytes and vascular endothelial cells (3, 12, 13). This direct viral invasion can initiate cellular injury. However, a predominant contributor to widespread cardiovascular damage is the dysregulated host immune response, culminating in a hyperinflammatory state often termed a “cytokine storm” (1, 13). This cascade of pro-inflammatory cytokines, such as interleukin-6 (IL-6) and tumor necrosis factor-alpha (TNF-α), leads to endothelial cell activation, inflammation, and dysfunction (13). Concomitantly, a pro-thrombotic milieu emerges, characterized by platelet activation, neutrophil-platelet aggregation, and a disruption of the fibrinolytic balance, fostering microvascular thrombosis and ischemic events (1, 11, 14). Other indirect mechanisms include hypoxia-induced myocardial injury, stress-related cardiomyopathy, and the downregulation of the protective ACE2 axis within the renin-angiotensin-aldosterone system (RAAS), further promoting inflammation and fibrosis (3, 15, 16). Despite this growing understanding of the clinical landscape and proposed mechanisms, the precise molecular orchestrators driving these interconnected pathological processes remain incompletely elucidated.

Non-coding RNAs (ncRNAs) have emerged as pivotal regulatory players. The human transcriptome is replete with ncRNAs, including microRNAs (miRNAs), long non-coding RNAs (lncRNAs), and circular RNAs (circRNAs), which traditionally classified as non-coding proteins but are fundamental regulators of gene expression at transcriptional, post-transcriptional, and epigenetic levels (17). Host ncRNAs display expression aberrations in the context of SARS-CoV-2 infection and are associated with viral infection dynamics, immune response modulation, and disease progression across multiple organ systems, including the heart and vasculature (17). They can influence critical pathways involved in inflammation, cell death (including apoptosis and pyroptosis), thrombosis, and endothelial integrity (9, 13). Moreover, the virus itself may exploit or manipulate host ncRNA networks to facilitate its replication or evade immune surveillance. This places ncRNAs not merely as observers but as active conductors in the pathological symphony of cardiac and vascular injury related to the SARS-CoV-2 infection.

The primary aim of this comprehensive review is to systematically summarizes current knowledge on the role of ncRNAs in the cardiovascular complications of COVID-19. First, we delineate the established pathophysiological mechanisms of SARS-CoV-2-induced cardiovascular damage, and provide a detailed landscape of the dysregulated ncRNA expression profiles associated with COVID-19 cardiac injury. Second, we explore the mechanistic insights of how these ncRNAs orchestrate key pathological events such as cardiac inflammation, programmed cell death, and thrombogenesis. The review will also discuss the emerging role of ncRNAs in mediating intercellular and systemic communication, particularly within the cardio-immune axis via extracellular vesicles. Finally, we will critically evaluate the translational potential of ncRNAs as novel diagnostic and prognostic biomarkers and as innovative therapeutic targets. The integration of evidence from the curated literatures is intended to facilitate the establishment of a comprehensive understanding of ncRNA biology in cardiovascular disease resulting from SARS-CoV-2 and to highlight promising avenues for future research and clinical application.

## 2 SARS-CoV-2 infection and cardiovascular pathophysiology: the stage for ncRNA dysregulation

**Figure 1:**
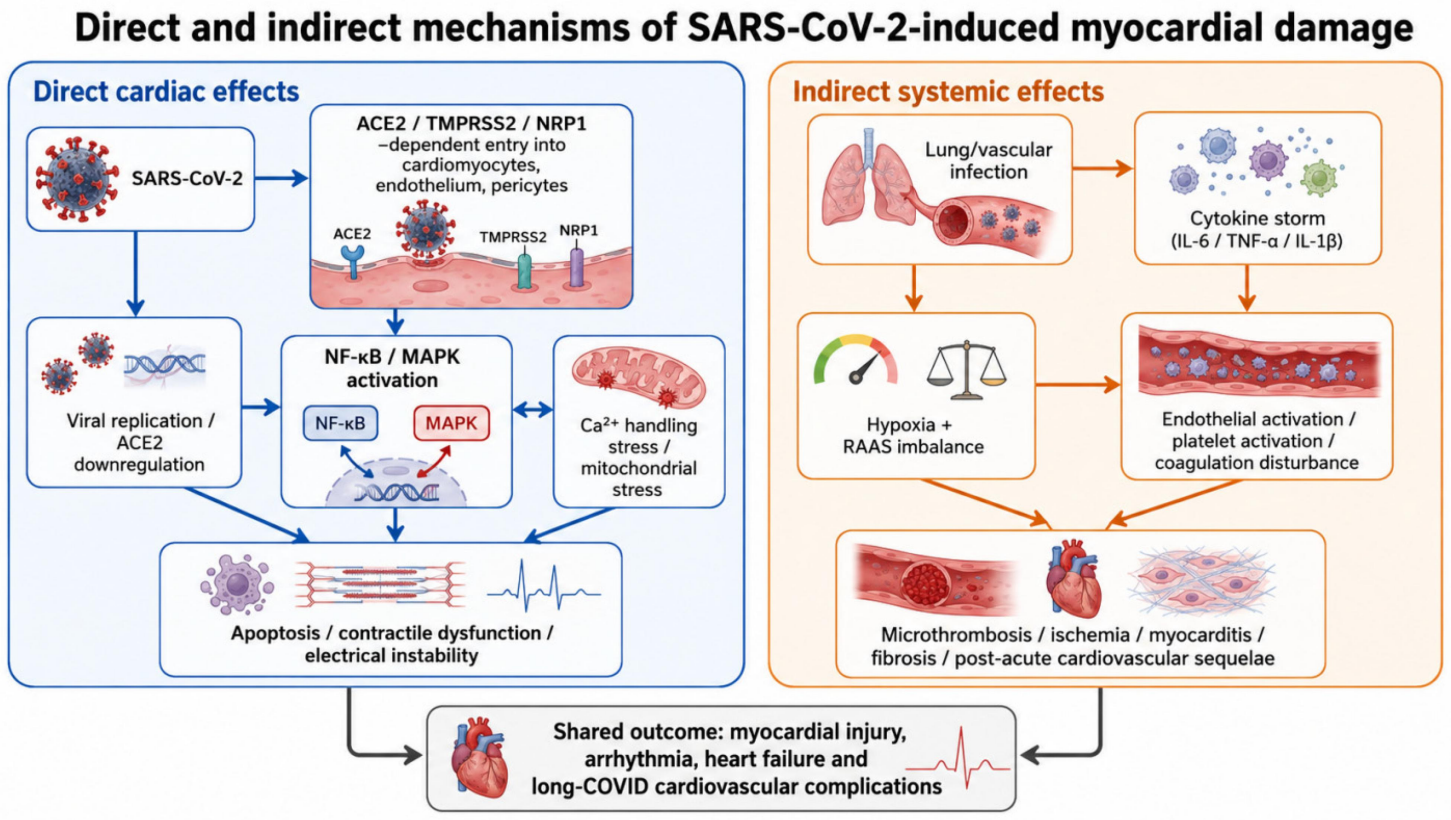
Direct and indirect mechanisms of SARS-CoV-2-induced myocardial damage. SARS-CoV-2 directly invades cardiovascular cells through ACE2/TMPRSS2/NRP1-dependent entry, inducing viral replication, ACE2 downregulation, NF-*κ*B/MAPK activation, mitochondrial and calcium-handling stress, apoptosis, contractile dysfunction, and electrical instability. Indirect injury is mediated by lung-derived systemic inflammation, cytokine storm, hypoxia, RAAS imbalance, endothelial activation, platelet activation, coagulation disturbance, microthrombosis, ischemia, myocarditis, fibrosis, and post-acute cardiovascular sequelae. The evidence suggests that both direct and indirect pathways lead to myocardial injury, arrhythmia, heart failure and long-term cardiovascular complications resulting from the condition. SARS-CoV-2, severe acute respiratory syndrome coronavirus 2; ACE2, angiotensin-converting enzyme 2; TMPRSS2, transmembrane serine protease 2; NRP1, neuropilin-1; NF-*κ*B, nuclear factor kappa B; MAPK, mitogen-activated protein kinase; RAAS, renin-angiotensin-aldosterone system; long-COVID, long coronavirus disease; IL-6, interleukin-6; TNF-α, tumor necrosis factor-alpha; IL-1β, interleukin-1 beta.

The intricate link between COVID-19 and cardiovascular complications is established upon a multifaceted pathophysiological stage, where SARS-CoV-2 orchestrates cardiac and vascular injury through a convergence of direct and indirect mechanisms (18, 19). This injury is present in a significant proportion of hospitalized patients and is a key factor in disease severity and mortality (20–22). The foundational event enabling this crosstalk is the interaction between the viral spike protein and the host ACE2 receptor, which is abundantly expressed not only in lung alveolar cells but also on cardiomyocytes, vascular endothelial cells, and cardiac pericytes (19, 20, 23). This widespread expression of ACE2, a key component of the renin-angiotensin system (RAS) counter-regulator*y* axis, positions the cardiovascular system as a potential target for viral invasion and systemic dysregulation (24–26). Upon entry into cardiomyocytes via surface ACE2 receptors, SARS-CoV-2 activates two critical signaling pathways: the NF-*κ*B pathway, which elicits inflammatory responses, and the MAPK pathway (27), which directly induces cardiomyocyte apoptosis. In addition to these direct effects, SARS-CoV-2-related lung injury can indirectly damage the heart through a number of mechanisms, including cytokine storm, hypoxia, RAAS imbalance, endothelial activation, platelet activation, and coagulation disturbance (15). The synergistic action of inflammation and apoptosis ultimately leads to myocardial damage (Figure 1).

Direct cardiac injury may occur when SARS-CoV-2 enters cardiomyocytes through ACE2, with the assistance of cofactors such as transmembrane protease serine 2 (TMPRSS2) and neuropilin-1 (NRP1) (28–30). The results of several autopsy studies have confirmed the presence of viral RNA and spike protein in myocardial tissue, supporting the possibility of direct myocardial involvement. SARS-CoV-2 infection of cardiac tissue may impair myocardial contractility, trigger electrical dysfunction, and activate apoptotic pathways (29, 31). In addition, viral binding to ACE2 can lead to ACE2 downregulation, thereby disrupting the cardioprotective ACE2/Ang- (1–7)/Mas axis and favoring excessive activation of the classical ACE/Ang II/AT1R axis. This imbalance may promote vasoconstriction, inflammation, oxidative stress, and fibrosis, ultimately exacerbating myocardial injury, hypertension, and pathological cardiac remodeling (32–35).

Concurrently, indirect cardiac injury may occur through a systemic hyperinflammatory state, often referred to as a “cytokine storm” (36–38). This process is characterized by the massive release of pro-inflammatory cytokines, including IL-6, TNF-α, and interleukin-1 beta (IL-1β) (39–41). This cytokine storm induces widespread endothelial dysfunction and endotheliitis, compromises vascular integrity, and establishes a pro-thrombotic state (42, 43). The activation of endothelial cells, platelets, and immunothrombosis collectively result in microcirculatory dysfunction, coronary microthrombosis, and plaque instability. The clinical manifestations of this process include acute coronary syndrome, myocardial infarction, or type 2 myocardial injury due to supply-demand mismatch (44–46). The coagulation cascade is further dysregulated, contributing to arterial and venous thromboembolism, a hallmark of severe COVID-19 (47).

Beyond direct viral and inflammatory effects, hypoxia caused by secondary respiratory failure, electrolyte disturbances, and elevated metabolic demand further exacerbate myocardial damage and precipitate or worsen arrhythmias and heart failure (48–50). Inflammatory infiltration of the myocardium can result in clinical or subclinical myocarditis, ranging from asymptomatic presentations to fulminant myocarditis complicated by cardiogenic shock (51). Other cardiovascular manifestations include stress-induced (Takotsubo) cardiomyopathy, pericarditis, and acute exacerbation of preexisting conditions such as heart failure and coronary artery disease (52–54). It is important to note that the aforementioned pathological changes are not confined to the acute phase, a considerable proportion of patients develop persistent cardiovascular abnormalities as part of post-acute sequelae of COVID-19 (PASC), or long COVID. These manifestations include persistent myocardial inflammation, fibrosis, endothelial injury, autonomic dysfunction, and an elevated long-term risk of major adverse cardiovascular events (55–58).

The propensity to experience cardiovascular complications related to COVID-19 varies considerably among individuals. Elderly individuals with preexisting cardiovascular conditions, including hypertension, diabetes, atherosclerosis, and heart failure, exhibit an elevated risk of myocardial injury (59, 60). This increased susceptibility is closely linked to preexisting ncRNA dysregulation in comorbidities, which alters the expression levels of key protective molecules even before infection. In hypertension, angiotensin II (Ang II) upregulates miR-122-5p, which suppresses the expression of ACE2, Apelin, and Elabela in cardiac tissue, creating a pro-fibrotic and pro-inflammatory baseline that worsens damage upon viral invasion (61). In diabetes, a high-glucose environment induces miR-125b expression, which directly binds to the 3′-UTR of ACE2 mRNA, decreasing ACE2 protein stability and promoting reactive oxygen species (ROS) generation and apoptosis (62). In addition, clinical cohort studies of COVID-19 have reported that downregulation of lncRNA growth arrest-specific 5 (GAS5). It may reduce its binding to miR-200, leading to increased miR-200 activity and suppression of ACE2 expression. This may weaken ACE2-mediated protective signaling and thereby exacerbate inflammatory injury (63). Concurrently, comorbidity-associated ncRNA alterations in COVID-19 patients showed not only target ACE2 but also act on multiple other targets, such as Apelin, and Elabela, thereby increasing vulnerability to COVID-19-associated cardiovascular injury. Nonetheless, the findings from these preliminary studies need further investigation in multicenter settings to confirm their potential value.

## 3 Landscape of non-coding RNA dysregulation in COVID-19-associated cardiovascular injury

SARS-CoV-2 infection disrupts the myocardial and vascular microenvironment, triggering dysregulation of host ncRNA expression profiles and thereby mediating a cascade of cardiovascular injuries. Such dysregulation mainly involves aberrant expression of miRNAs, lncRNAs, and circRNAs, as well as regulatory effects of virally encoded ncRNAs, collectively forming a complex regulatory network that influences cardiovascular outcomes in COVID-19 (64, 65).

miRNAs have been most extensively studied, revealing a distinct pattern of circulating and tissue-specific dysregulation linked to disease severity and specific cardiovascular complications (66, 67). For instance, clinical and bioinformatic data reveal that miR-155-5p is significantly elevated in hospitalized COVID-19 patients. It represses target gene suppressor of cytokine signaling 1 (SOCS1), thereby driving the cytokine storm and aggravating vascular endothelial damage (68). Concurrently, the thrombosis-related miR-16-5p and miR-27a-3p promote microthrombus formation by modulating coagulation target genes such as fibronectin 1 (FN1) and epidermal growth factor receptor (EGFR) (69). In the context of myocardial injury, the upregulation of miRNA-146a in severe patients has been demonstrated to target and suppress the cyclin-dependent kinase inhibitor 1B (CDKN1B). This dysregulation has been shown to be closely correlated with abnormal elevations of the heart failure marker, proBNP (70). However, the interaction of miRNAs and targets based on computational analysis need experimental validation, such as reporter assay. Further investigation in larger patient cohorts is necessary to understand the role of these miRNAs in the progression of viral infections.

Beyond miRNAs, dysregulated lncRNAs also play an important role in COVID-19-related cardiovascular injury. Studies on competing endogenous RNA (ceRNA) networks have shown that lncRNAs or circular RNAs often function as “molecular sponges” to sequester specific miRNAs, thereby modulating the expression of downstream target genes. This “lncRNA-miRNA-target gene” cascade helps explain how viral infections contribute to cardiovascular pathology (71). For instance, the lncRNA MALAT1 is closely associated with COVID-19-related endothelial dysfunction (72). Clinical and cellular studies indicate that MALAT1 levels decrease during SARS-CoV-2 infection, which may impair its ability to effectively sponge miR-200c-3p. The resulting increase in free miR-200c-3p subsequently suppresses the target gene SIRT1, a crucial player in vasoprotection and anti-inflammation, potentially exacerbating endothelial injury and inflammatory responses in patients (73). Similarly, in heart failure models, the reduction of lncRNA cytb in damaged myocardium releases its bound miR-103-3p, leading to the inhibition of the downstream target gene phosphatase and tensin homolog (PTEN). Conversely, moderately restoring lncRNA cytb levels helps reactivate the PTEN target gene, thereby alleviating cardiomyocyte oxidative stress and pathological hypertrophy to some extent (74). However, causal evidence directly linking specific lncRNAs to COVID-19-induced cardiovascular damage remains limited. These findings suggest that evaluating lncRNA dysregulation should not be restricted solely to alterations in their own expression levels. Rather, objectively dissecting the potential mechanisms of cardiovascular injury requires linking these non-coding transcripts directly to the specific target genes.

circRNAs are covalently closed-loop RNA molecules that primarily regulate gene expression through miRNA sponging, interactions with RNA-binding proteins, and modulation of innate immune signaling (75, 76). During COVID-19, both host-derived circular RNAs and SARS-CoV-2-encoded circular RNAs may be involved in antiviral responses, inflammation, oxidative stress and host-virus interactions, suggesting that viral circular RNAs are not merely transcriptional by-products but potential regulators of the pathogenesis of SARS-CoV-2 (77). For instance, *in vitro* experiments on endothelial cells show that circSARS-CoV-2-N1368, derived from the viral nucleocapsid gene, can specifically bind to host miR-103a-3p. The present study demonstrates that this binding removes the suppression on the host target gene, activating transcription factor 7 (ATF7), and activates the TLR4/NF-*κ*B signaling pathway, thereby driving the production of ROS. This, in turn, causes oxidative damage in endothelial cells (78). It is important to note that the present study is currently limited in its ability to provide definitive validation, as it has not been further substantiated in animal models or clinical cohorts. Consequently, at this stage, the study primarily functions as an emerging mechanistic hint. Similarly, the host-derived hsa_circ_0000479 is significantly upregulated and competitively binds to hsa-miR-149-5p to release the inhibition on retinoic acid-inducible gene I (RIG-I), which activates downstream IL-6 expression and forms a circRNA-miRNA-mRNA regulator*y* axis involved in the immune response (79). Furthermore, in the context of COVID-19, circRasGEF1B and circHIPK3 are upregulated in peripheral blood mononuclear cells (PBMCs) (80). circRNA sequencing of peripheral blood (from 3 patients with recurrent COVID-19 and 3 healthy controls) identified multiple differentially expressed circRNAs. However, due to the extremely small sample size and the lack of validation correlating them with heart injury markers like cardiac troponin I (cTnI), this remains an exploratory bioinformatics study (81). While studies based on animal models of heart injury indicate that abnormally high circHIPK3 can competitively bind to miR-185-3p to upregulate the calcium-sensing receptor (CASR) target gene, further worsening heart cell death and inflammatory infiltration (82), this remains an indirect link to COVID-19-specific heart injury and has not yet been confirmed in large COVID-19 patient cohorts. Therefore, circRNA-mediated gene regulatory networks may be regard as a potential direction for future research.

## 4 Mechanistic insights: potential involvement of ncRNAs in cardiac inflammation, cardiomyocyte apoptosis, and thrombosis

**Figure 2:**
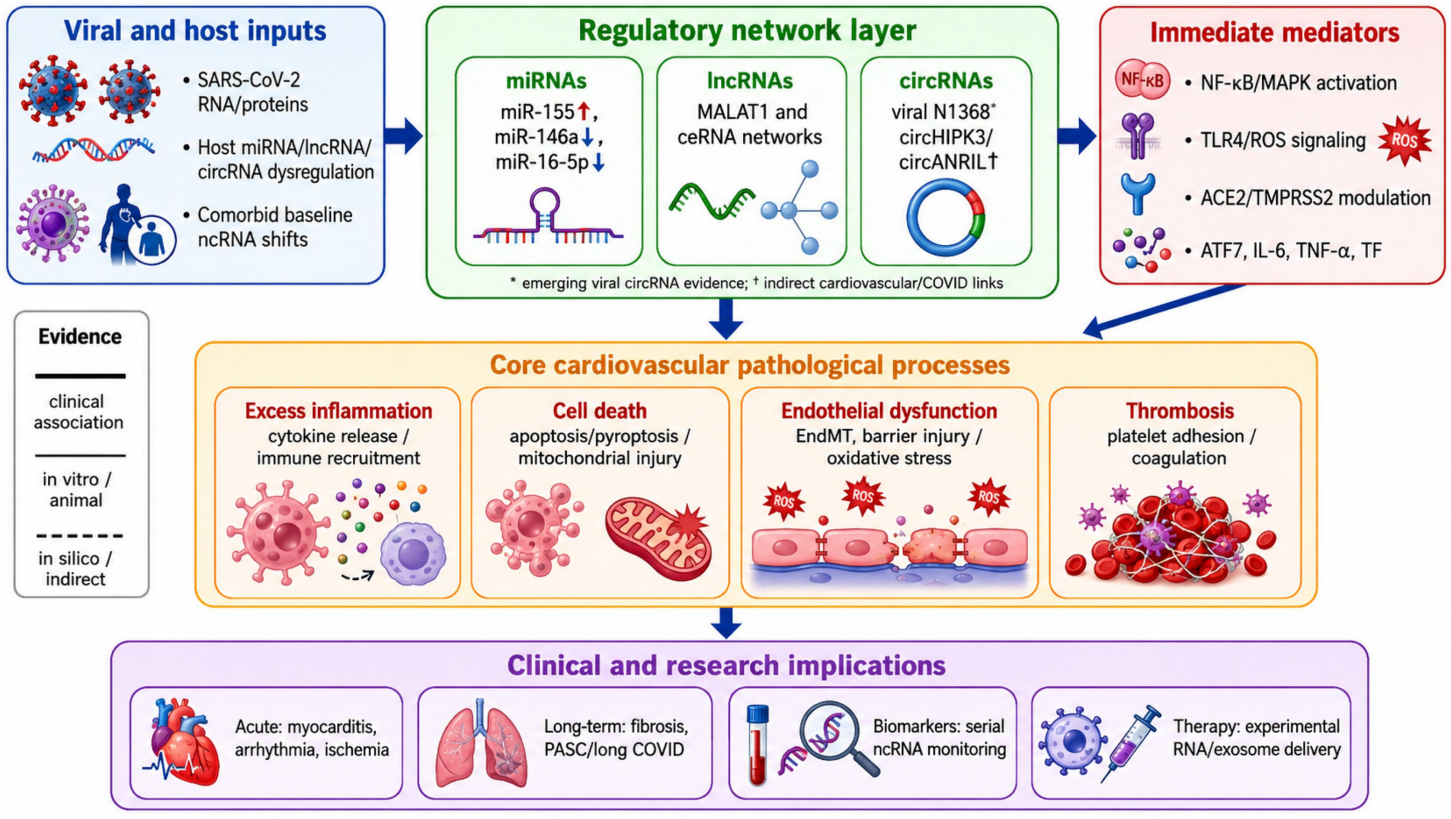
ncRNA-coordinated cardiac inflammation, cell death, endothelial dysfunction, and thrombosis in COVID-19-associated cardiovascular injury. The figure links viral and host inputs to three ncRNA regulatory layers (miRNAs, lncRNAs, and circRNAs), downstream mediators (NF-*κ*B/MAPK, TLR4/ROS, ACE2/TMPRSS2 modulation, ATF7, IL-6, TNF-α and tissue factor), core pathological processes, and acute/long-term cardiovascular outcomes. The upward arrow indicates upregulated expression, and the downward arrow indicates downregulated expression. ncRNA, non-coding RNA; miRNAs, microRNAs; lncRNAs, long non-coding RNAs; circRNAs, circular RNAs; NF-*κ*B, nuclear factor kappa B; MAPK, mitogen-activated protein kinase; TLR4, toll-like receptor 4; ROS, reactive oxygen species; ACE2, angiotensin-converting enzyme 2; TMPRSS2, transmembrane serine protease 2; ATF7, activating transcription factor 7; IL-6, interleukin-6; TNF-α, tumor necrosis factor-alpha; endothelial-mesenchymal transition (EndMT); circHIPK3, circular homeodomain-interacting protein kinase 3; circANRIL, circular antisense non-coding RNA in the INK4 locus.

Dysregulated ncRNAs may play a role in four main processes of COVID-19 cardiovascular injury: cardiac inflammation, cell death, endothelial dysfunction, and thrombosis (Figure 2). Cardiac inflammation is the core driver of this cardiac damage. The viral infection might not only damage the heart directly but also worsen the condition by disrupting the host's ncRNA networks and affecting specific gene expression (83). By using *in vitro* cell and animal models, studies found that modified mRNAs, which mimic the components of COVID-19 mRNA vaccines, can also act as ceRNAs to bind host hsa-let-7f-5p in cardiomyocytes, thereby increasing IL-6 secretion and triggering cell death (71). However, these findings should be interpreted with caution, as there are conflicting conclusions across different cohort studies. For instance, clinical studies employing blood samples have demonstrated that the anti-inflammatory microRNA-146a-5p, which regulates inflammatory target genes such as interleukin-1 receptor-associated kinase 1 (IRAK1) and TNF receptor-associated factor 6 (TRAF6), exhibits a significant increase in certain patient populations (84). In other patient groups with severe cases or preexisting conditions, its levels undergo a substantial decrease, resulting in the loss of control of these target genes and the exacerbation of inflammation (85). The observed variability may be attributable to that patient age, preexisting health conditions, and the temporal context of sample collection can exert a significant influence on research results. The core of risk stratification lies in the early identification of high-risk patients who may progress to fulminant myocarditis or malignant arrhythmias. Moving beyond minor changes within individual cells, the altered expression of ncRNAs in PBMCs could more directly reflect the connection between host immune overactivation and cardiovascular events, making them useful for early patient risk stratification.

Concurrently, SARS-CoV-2 causes cardiomyocyte apoptosis and mitochondrial damage, with ncRNAs acting as key molecules that disrupt cellular metabolism during this process. For instance, SARS-CoV-2 infection has been observed to abnormally activate host miRNA-2392, which has been shown to target and post-transcriptionally inhibit translocase of outer mitochondrial membrane 20 (TOMM20), a key mitochondrial outer membrane protein gene. Then, it has been observed to trigger a substantial release of ROS, ultimately driving cardiomyocytes towards a state of apoptosis (86). However, given the study's reliance on a modest clinical sample size, validation in larger cohorts is necessary to ensure generalizability of the findings. Additionally, the *in vivo* experiments were conducted exclusively in hamsters, highlighting the need for further evaluation of the safety and effectiveness of the treatment in humans.

Endothelial dysfunction is a key feature of COVID-19-associated cardiovascular injury, with miRNAs potentially regulating endothelial-mesenchymal transition (EndMT), barrier integrity, oxidative stress, and thrombotic processes. For example, miR-1-3p, verified in clinical samples, targets Krüppel-like factor 4 (KLF4), transforming growth factor beta receptor 1 (TGF*β*R1), and plasminogen activator inhibitor-1 (PAI-1) to induce endothelial-mesenchymal transition and thrombosis. Its plasma level predicts disease severity and prognosis in hospitalized COVID-19 patients (87). miRNA-126 has been identified as a key regulator of endothelial barrier maintenance and thrombosis inhibition. This regulatory function of miRNA-126 involves the targeting of sprouty-related EVH1 domain-containing protein 1 (SPRED1), phosphoinositide-3-kinase regulatory subunit 2 (PIK3R2), and vascular cell adhesion molecule 1 (VCAM-1) (88). This study enrolled 40 COVID-19 patients and 10 healthy controls. Reduced miR-126 expression was closely associated with severe COVID-19 and an increased risk of venous thrombosis (89). In addition, exosomes derived from human induced pluripotent stem cells (iPSCs) alleviate SARS-CoV-2-induced endothelium-myocardial injury via miR-19a-3p and miR-20a-5p. The present study identifies PTEN, autophagy-related 7 (ATG7) and tumor protein p53 (TP53) as potential downstream targets. The findings of this study suggest that iPSC-EVs have the capacity to protect against SARS-CoV-2 infection by delivering exosomes derived miRNAs to recipient cells (90). Together, we cannot simply treat the effects observed *in vitro* experiments or small clinical cohorts as the definitive mechanisms driving cardiovascular injury, and future studies will require more rigorous evidence.

A further limitation is that many ncRNA studies rely on a single timepoint, whereas COVID-19 evolves dynamically through viral replication, immune activation, endothelial injury, and thromboinflammation. This suggests that the expression patterns of ncRNAs might also change alongside these processes, thereby affecting the development of cardiovascular complications. For example, the SARS-CoV-2 source circ_3205 correlates positively with Spike mRNA and viral load, and may regulate coagulation- and immune-related genes such as KCNMB4 and PRKCE via miR-298, suggesting a potential link between rising viral replication and subsequent immune-thrombotic responses (91). A longitudinal RNA sequencing analysis comparing patients in their early and late stages found that dynamic changes in certain circRNAs might help distinguish COVID-19 survivors from non-survivors (92). Meanwhile, another analysis further suggests that as COVID-19 infection worsens, the dynamic dysregulation of specific circRNAs, such as hsa_circ_0080942 and hsa_circ_0080135, might indirectly regulate immune-related pro-inflammatory target genes, thereby contributing to systemic inflammation and subsequent cardiovascular injury (93). These findings indicate that continuously monitoring the dynamic changes of ncRNAs and their target genes can help us better understand the overall progression of the disease. However, it should be acknowledged that large-scale, longitudinal tracking studies focusing specifically on cardiovascular-specific ncRNAs are still lacking. In summary, dysregulated ncRNAs may contribute to key pathological processes in COVID-19-associated cardiovascular injury (Table 1).

**Table 1:** Regulatory mechanisms and functions of key non-coding RNAs in COVID-19-associated cardiovascular injury.

Non-coding RNA type	Specific molecule name	Main mechanism/target	Pathological consequences	Evidence source
miRNA	miR-122-5p↑	Ang II-induced miR-122-5p suppresses ACE2, apelin and elabela in cardiac tissue.	pro-fibrotic/pro-inflammatory comorbidity that may worsen viral injury.	Animal evidence; comorbidity-related, indirect COVID-19 relevance (61).
miRNA	miR-125b↑	High glucose induces miR-125b, which binds the 3′-UTR of ACE2 mRNA and reduces ACE2 stability.	Promotes ROS generation and apoptosis, potentially increasing vulnerability in diabetes.	*In vitro* evidence; diabetes/comorbidity model (62).
miRNA	miR-155-5p↑	Targets SOCS1 and amplifies inflammatory signaling.	Drives cytokine-storm responses and aggravates vascular endothelial damage.	Clinical + in silico evidence (68).
miRNA	miR-16-5p/miR-27a-3p	Modulate coagulation-related genes including FN1 and EGFR.	Promote microthrombus formation and thrombosis-related cardiovascular injury.	Clinical + in silico evidence (69).
miRNA	miR-133a	miR-133a dynamics relate to short-term prognosis.	Potentially contributes to cardiac remodeling, fibrosis, arrhythmia and post-acute outcomes.	Clinical longitudinal evidence; specificity remains limited (100).
miRNA	miR-2392↑	Post-transcriptionally inhibits TOMM20 and disrupts mitochondrial integrity.	Promotes ROS release, mitochondrial dysfunction and cardiomyocyte apoptosis.	Clinical + in silico + *in vitro* evidence (86).
miRNA	miR-1-3p↑/miR-126↓	miR-1-3p targets KLF4, TGF*β*R1 and PAI-1; miR-126 regulates SPRED1, PIK3R2 and VCAM-1.	Links endothelial dysfunction to thrombosis, disease severity and prognosis in hospitalized patients.	Clinical evidence; miR-126 cohorts remain relatively small (87–89).
miRNA	miR-19a-3p/miR-20a-5p	iPSC-derived exosomal miRNAs may regulate PTEN, ATG7 and TP53.	May alleviate SARS-CoV-2-induced endothelium-myocardial injury.	In silico + *in vitro* evidence; *in vivo* and large clinical validation lacking (90).
lncRNA	miR-200 axis	GAS5 downregulation releases miR-200, leading to excessive ACE2 inhibition.	May exacerbate inflammatory injury and severe COVID-19 in patients with comorbid dysregulation.	Clinical cohort evidence (63).
lncRNA	MALAT-1/MALAT1↓	Reduced MALAT1 weakens miR-200c-3p sponging, allowing suppression of SIRT1.	Weakens vasoprotective, anti-inflammatory and anti-apoptotic defenses; aggravates endothelial injury.	Clinical + *in vitro* evidence (73); causal COVID-cardiac evidence remains limited.
lncRNA	lncRNA cytb↓	Reduced lncRNA cytb releases miR-103-3p and inhibits PTEN.	Increases cardiomyocyte oxidative stress and pathological hypertrophy in heart-failure models.	Animal/cardiovascular model evidence; indirect link to COVID-19 cardiac injury (74).
lncRNA	LEF1-AS1/HCG18/H19/TUG1/CRNDE	PBMC lncRNA dysregulation correlates with immune activation, NLR, STAT3 and *α*-SMA-related fibrosis signatures.	May support risk stratification for severe/fatal COVID-19 and inflammatory cardiovascular risk.	Clinical blood-sample + *in vitro* evidence; indirect cardiovascular specificity (102, 103).
circRNA	circSARS-CoV-2-N1368	Viral circRNA sponges miR-103a-3p, releases ATF7, and activates TLR4/NF-*κ*B/ROS signaling.	Causes endothelial oxidative damage and may promote prothrombotic vascular dysfunction.	*In vitro* evidence only; no animal or clinical validation, emerging mechanistic clue (78).
circRNA	hsa_circ_0000479	Competitively binds hsa-miR-149-5p, releases RIG-I and activates IL-6 signaling.	May amplify antiviral immune signaling and IL-6-driven inflammatory injury.	*In vitro* evidence with circRNA-miRNA-mRNA axis; COVID cardiovascular link indirect (79).
circRNA	circRasGEF1B/circHIPK3	Upregulated in COVID-19 PBMCs; circHIPK3 can sponge miR-185-3p to upregulate CASR in heart-injury models.	Potential immune-regulatory biomarkers; circHIPK3 may aggravate cell death and inflammatory infiltration.	COVID clinical-sample association + animal/*in vitro* cardiovascular evidence; direct COVID-cardiac evidence lacking (80).
circRNA	circ_3205	SARS-CoV-2-derived circ_3205 correlates with Spike mRNA and viral load and may regulate KCNMB4 and PRKCE via miR-298.	May link viral replication with coagulation- and immune-related responses and immune-thrombotic injury.	In silico/*in vitro* evidence; cardiovascular link remains indirect (91).
circRNA	hsa_circ_0080942/hsa_circ_0080135	Dynamic dysregulation may regulate immune-related pro-inflammatory target genes during disease progression.	Supports longitudinal circRNA monitoring for outcome stratification and inflammatory progression.	In silico evidence; cardiovascular-specific longitudinal studies lacking (93).

## 5 Intercellular and systemic communication mediated by ncRNAs in the cardio-immune axis

ncRNA-mediated regulatory networks are not limited to individual cells but may also extend across diverse cell types and organ systems. These networks serve as pivotal mediators, orchestrating a complex set of interactions that are central to the cardiovascular pathologies associated with SARS-CoV-2. In ncRNAs have been observed to employ extracellular vesicles (EVs) to facilitate communication between cardiomyocytes, endothelial cells, and local immune cells, including both resident and infiltrating macrophages (94). During the cardiac injury triggered by SARS-CoV-2 infection, both the activation state of macrophages and the contents of their exosomes undergo changes. Studies showed that pro-inflammatory macrophages can interfere with cardiomyocyte function by releasing exosomes rich in specific ncRNAs. For example, abnormally expressed miRNAs in these exosomes can induce cell death or disrupt the electrical signaling in heart cells (95). Meanwhile, damaged cardiomyocytes also release vesicles containing specific damage-related ncRNAs into their surrounding environment. This further recruits and activates monocytes and macrophages, creating a local and ongoing cycle of inflammation.

ncRNA-mediated communication goes beyond single-organ boundaries, serving as a key link that connects the primary lung infection, the systemic immune storm, and distant cardiovascular damage. Severe COVID-19 causes changes in both the profiles of circulating EV-miRNAs and the internal ncRNAs of blood cells, such as red blood cells and lymphocytes (96). For instance, specific miRNAs, like miR-199a-5p and miR-200a-3p, are significantly elevated in the circulating EVs of severe patients. Released by infected or overactivated immune cells, these vesicles are not just markers of host immune dysregulation. They can also travel through the bloodstream into the heart, allowing abnormal systemic immune signals to directly impact the cardiovascular system (97). Furthermore, some circulating miRNAs that normally provide anti-inflammatory protection in the cardiovascular system are suppressed after infection. For example, the downregulation of miR-374a in severe COVID-19 patients may removes the post-transcriptional suppression of inflammatory signaling, including the IL-6R/CCL2 axis, thereby amplifying the cytokine storm and increasing the risk of secondary cardiac injury (98).

In summary, COVID-19-related cardiovascular damage may be influenced by ncRNA-mediated intercellular and cell-to-cell communication. During this process, ncRNA-mediated communication within the cardiovascular-immune axis works synergistically across different spatial levels to drive the disease: local cell-to-cell communication worsens immune infiltration and heart cell damage within the cardiac microenvironment, while systemic communication delivers inflammatory signals from the primary infection site to the cardiovascular network. Together, these two processes likely contribute to multi-organ inflammatory syndrome and cardiovascular dysfunction.

## 6 Translational applications: ncRNAs as biomarkers and therapeutic targets

Among cardiovascular injuries associated with COVID-19, the altered expression profiles of ncRNAs hold potential clinical value and may serve as biomarkers for disease diagnosis, risk stratification, and prognosis assessment (66, 99). However, to translate these findings from the preliminary exploratory stage to practical clinical application, a methodological evaluation, performance validation, and multicenter clinical trials are necessary to assess their feasibility as diagnostic tools. In the early diagnosis of disease, circulating miRNAs can sensitively reflect damage within the cardiac microenvironment. For instance, analyses based on large-scale clinical blood samples show that miRNAs such as miR-146a, miR-451, and miR-21 are specifically dysregulated in severe COVID-19 patients. Notably, this distinct cardiac miRNA expression profile can distinguish COVID-19 patients from those with acute respiratory distress syndrome (ARDS) caused by influenza (67). In another prospective cohort, the abnormal expression of miR-133a and miR-122 was linked to heart muscle injury. Since these miRNAs can be observed elevated in skeletal muscle injury, liver damage, and sepsis, their specificity for diagnosing COVID-19 myocarditis alone is limited (100).

Studies have found that the expression levels of the lncRNAs LEF1-AS1 and HCG18 in peripheral blood are reduced in severe and fatal COVID-19 cases, and this reduction negatively correlates with the neutrophil-to-lymphocyte ratio (NLR) (101). Similarly, key lncRNAs (such as H19, TUG1, and CRNDE) are dysregulated in the PBMCs of COVID-19 patients and are co-expressed with markers for inflammation and cardiovascular fibrosis, such as signal transducer and activator of transcription 3 (STAT3) and alpha-smooth muscle actin (*α*-SMA) (102). Huang et al. performed an integrated single-cell sequencing analysis and identified differential expression of NEAT1 and MALAT1 in patients with severe COVID-19. Nevertheless, their findings were derived from retrospective data mining and have yet to be validated using independent clinical specimen cohorts (103).

For evaluating long-term cardiovascular outcomes (such as heart failure or recurrent microthrombosis caused by Long COVID), circRNAs are highly resistant to exonucleases due to their unique covalently closed circular structure. This structure grants them a much longer half-life in plasma and tissues compared to linear RNAs (104). Studies indicate that within the cardiovascular system, specific circRNAs are involved in the development of ischemic heart disease, cardiac fibrosis, and cardiomyopathy. Their lasting changes in expression make them promising tools for long-term prognostic monitoring (105). Heydari et al. used machine learning methods to screen public databases for COVID-19-related lncRNA and circRNA. However, because this study relied on bioinformatics analysis, it still lacks validation in independent clinical cohorts (102). Furthermore, there is a clear shortage of dynamic monitoring and long-term follow-up data for prognostic assessment. For example, although longitudinal sampling showed that dynamic changes in miR-133a correlate with 28-day prognosis, the follow-up endpoint was limited to short-term ICU mortality and did not cover long-term outcomes like post-discharge heart failure, hospital readmission, or new-onset arrhythmias (100). Table 2 summarizes the potential applications of ncRNAs and the key challenges that may affect their clinical use.

**Table 2:** Translational application prospects of Non-coding RNAs in COVID-19 cardiovascular complications.

Application field	Specific strategy/molecule	Clinical value and function	Potential challenges
Diagnostic biomarkers	Cardiac/severity miRNA panel: miR-146a, miR-451 and miR-21	May help distinguish severe COVID-19-related cardiac injury signatures from influenza-associated ARDS.	Requires standardized sampling, normalization and validation beyond systemic inflammation (67).
Diagnostic biomarkers	miR-133a and miR-122	Associated with myocardial injury in prospective clinical cohorts.	Specificity is limited because skeletal muscle injury, liver damage and sepsis can also elevate these miRNAs (100).
Exploratory biomarkers	Peripheral-blood circRNA sequencing	May identify recurrent-COVID circRNA signatures and long-lasting regulatory changes.	Extremely small discovery cohorts and limited correlation with cTnI or cardiovascular outcomes (81).
Risk stratification	LEF1-AS1, HCG18, H19, TUG1 and CRNDE	PBMC lncRNA dysregulation may reflect immune activation, NLR changes, and STAT3/α-SMA-related inflammatory-fibrotic signatures.	Needs multicenter validation, independent clinical samples and integration with cardiovascular endpoints (101, 102).
Algorithm-based prediction	NEAT1 and MALAT1	Single-cell/data-mining analyses may help identify severe COVID-19 immune-endothelial signatures.	Data-mining results require independent clinical-sample validation before routine use (103).
Long-term prognosis	Stable circRNA signatures	Because circRNAs resist exonucleases, they may support monitoring of Long-COVID cardiovascular risk, fibrosis or cardiomyopathy.	Need longitudinal cardiovascular cohorts and long-term endpoints beyond acute ICU mortality (104).
Therapeutic targets	ASOs/siRNA against pathogenic ncRNA axes	Theoretically suppresses excessive inflammation, fibrosis or prothrombotic signaling.	No late-stage ncRNA-targeted trials for COVID-19 cardiovascular injury; off-target and immunogenicity risks remain major barriers (113).
Therapeutic restoration	miRNA mimics or protective exosomal miRNAs	May restore protective miRNA functions, such as iPSC-exosome miR-19a-3p/miR-20a-5p effects on endothelium-myocardial injury.	Dose control, biodistribution, durability and large-scale *in vivo*/clinical validation remain unresolved (90).

ncRNA has emerged as a promising therapeutic tool against SARS-CoV-2. Gasparello et al. reported that bronchial epithelial IB3-1 cells by treatment with the SARS-CoV-2 Spike protein could reduce IL-8 synthesis and extracellular release by using an agomiR molecule mimicking miR-93-5p (106). Presently, the majority of therapeutic approaches prioritize the suppression of pathogenic lncRNAs through the delivery of synthetic lncRNAs. A variety of methodologies exist for the delivery of lncRNA. These include the use of adeno-associated viruses, herpesviruses, ncRNA sponges, and so on (107). Pfafenrot et al. developed a series of antisense-circRNAs targeted to the SARS-CoV-2 genome RNA to inhibit viral replication by blocking the SARS-CoV-2 5′ UTR *in vitro* (108). Recently, some studies reported the successful use of preparing *in vitro* transcription (IVT) -derived circRNA for vaccination against SARS-CoV-2 (109, 110). However, as with any emerging technology, circRNA therapeutics have several limitations that must be addressed prior to their widespread adoption. The process of preparing IVT circular RNA in a scalable and reproducible manner is technically challenging. In addition, the development of effective tools for impurity removal is imperative, as this ensures low immunogenicity and good safety *in vivo* (110).

## 7 Challenges, future directions, and integration with emerging technologies

Translating ncRNA precision medicine from mechanistic discovery to clinical application for COVID-19 cardiovascular injury still faces many practical challenges. First, as biomarkers, existing ncRNAs often struggle to distinguish specific heart injury signals caused by COVID-19 from systemic inflammation or the complications of a patient's preexisting heart conditions (111). Additionally, the best timing for collecting samples to predict acute events and long-term consequences remains undetermined (112). In terms of therapeutic delivery, effectively and specifically targeting RNA drugs to the heart while avoiding off-target effects and adverse reactions remains a major research hurdle (113, 114). Furthermore, while the high stability of certain ncRNAs (like circRNAs) can prolong drug efficacy, it also raises concerns about how controllable these external treatments will be in the long run (115). At the same time, clarifying the potential ceRNA crosstalk mechanisms behind rare adverse events, such as mRNA vaccine-related myocarditis, is crucial for designing safer intervention strategies (116).

To address the lingering cardiovascular symptoms of Long COVID, future research must shift toward combining systems biology with multi-omics approaches. For Long COVID patients experiencing persistent symptoms like palpitations and shortness of breath upon exertion, exploring specific circulating ncRNA profiles might provide highly valuable molecular clues regarding microvascular dysfunction or hidden heart inflammation (117). To explore these pathological processes, future studies can use single-cell and spatial transcriptomics technologies to determine the specific cell types during ncRNA network dysregulation (118). If these findings can be cross-referenced with proteomic and metabolomic data to identify hub targets that connect multiple disease pathways, it could provide more reasonable entry point for future clinical treatments (119).

The clinical application of ncRNAs also depends on deep integrating with emerging technologies, such as artificial intelligence (AI) and dynamic data streams. Using AI algorithms, researchers can try to merge dynamically changing ncRNA data with multiple sources of information, including cardiovascular imaging, patient clinical traits, and genetic features, to build personalized, dynamic risk prediction models (120, 121). This approach could improve risk assessment and clinical monitoring in high-risk patients. Furthermore, integrating daily physiological data from wearable devices holds the potential to create a much more continuous early warning system (120). However, most current candidate ncRNA markers and AI models are still in their early stages. Ultimately, they must undergo rigorous testing through large, multicenter research networks and high-quality biobanks to truly cross the gap from early discovery to routine clinical use.

## 8 Conclusion and perspectives

The pathophysiological interaction between SARS-CoV-2 infection and the cardiovascular system is a complex cascade involving direct viral damage, immune and inflammatory dysregulation, endothelial dysfunction, and a pro-thrombotic state, all of which can lead to both acute heart injury and long-term sequelae (122). ncRNAs, including miRNAs, lncRNAs, and circRNAs, may regulate multiple aspects of COVID-19-associated cardiovascular injury, including viral entry and replication, inflammatory responses, apoptosis, pyroptosis, endothelial activation, and platelet aggregation. Furthermore, extracellular vesicle-associated ncRNAs may mediate intercellular and systemic communication within the cardio-immune axis (66). Their dynamic expression patterns are influenced by infection status and may, in turn, modulate host responses, forming feedback loops that could either aggravate tissue injury or contribute to repair processes (17).

This mechanistic research holds translational value. On one hand, ncRNAs show potential as novel biomarkers for the early detection of myocardial injury, risk stratification, and the prognostic assessment of acute and chronic cardiovascular complications (66). On the other hand, with the development of RNA therapies, using tools like antisense oligonucleotides (ASOs) or siRNAs to intervene in specific pathological pathways, such as excessive inflammation or fibrosis, offers a potential strategy for treating cardiovascular damage (113). However, clinical translation still faces many challenges: the heterogeneity of patient populations, the dynamic changes of ncRNA expression during disease progression, and the presence of preexisting comorbidities all require rigorous validation in large longitudinal cohorts (112), and the standardization of sample collection and data analysis techniques is urgently needed. Furthermore, how to safely and efficiently deliver RNA to specific heart cells via nanoparticle or exosome systems remains a major hurdle in current pharmacological research.

Long COVID patients often exhibit persistent cardiopulmonary symptoms (such as autonomic dysfunction or reduced exercise tolerance) despite having no obvious structural cardiac injury. Tracking their long-term ncRNA expression changes may help reveal the molecular mechanisms underlying these symptoms (123). Relying on standardized clinical biobanks to screen and validate ncRNA targets that regulate multiple key pathological pathways will be the core task ahead. Overall, studies of ncRNAs in SARS-CoV-2 infection may help bridge molecular mechanisms and clinical manifestations of cardiovascular disease. Further advances in methodology, biomarker validation, and delivery technologies are needed before these findings can provide a reliable basis for the diagnosis and treatment of COVID-19-associated cardiovascular sequelae.
